# The “Missing 6 dB” Revisited: Influence of Room Acoustics and Binaural Parameters on the Loudness Mismatch Between Headphones and Loudspeakers

**DOI:** 10.3389/fpsyg.2021.623670

**Published:** 2021-03-26

**Authors:** Florian Denk, Michael Kohnen, Josep Llorca-Bofí, Michael Vorländer, Birger Kollmeier

**Affiliations:** ^1^Medizinische Physik and Cluster of Excellence “Hearing4all”, Universität Oldenburg, Oldenburg, Germany; ^2^Institute of Technical Acoustics, RWTH Aachen University, Aachen, Germany

**Keywords:** psychoacoustics, headphone calibration, binaural loudness summation, room acoustics, cross site comparison

## Abstract

Generations of researchers observed a mismatch between headphone and loudspeaker presentation: the sound pressure level at the eardrum generated by a headphone has to be about 6 dB higher compared to the level created by a loudspeaker that elicits the same loudness. While it has been shown that this effect vanishes if the same waveforms are generated at the eardrum in a blind comparison, the origin of the mismatch is still unclear. We present new data on the issue that systematically characterize this mismatch under variation of the stimulus frequency, presentation room, and binaural parameters of the headphone presentation. Subjects adjusted the playback level of a headphone presentation to equal loudness as loudspeaker presentation, and the levels at the eardrum were determined through appropriate transfer function measurements. Identical experiments were conducted at Oldenburg and Aachen with 40 normal-hearing subjects including 14 that passed through both sites. Our data verify a mismatch between loudspeaker and binaural headphone presentation, especially at low frequencies. This mismatch depends on the room acoustics, and on the interaural coherence in both presentation modes. It vanishes for high frequencies and broadband signals if individual differences in the sound transfer to the eardrums are accounted for. Moreover, small acoustic and non-acoustic differences in an anechoic reference environment (Oldenburg vs. Aachen) exert a large effect on the recorded loudness mismatch, whereas not such a large effect of the respective room is observed across moderately reverberant rooms at both sites. Hence, the non-conclusive findings from the literature appear to be related to the experienced disparity between headphone and loudspeaker presentation, where even small differences in (anechoic) room acoustics significantly change the response behavior of the subjects. Moreover, individual factors like loudness summation appear to be only loosely connected to the observed mismatch, i.e., no direct prediction is possible from individual binaural loudness summation to the observed mismatch. These findings – even though not completely explainable by the yet limited amount of parameter variations performed in this study – have consequences for the comparability of experiments using loudspeakers with conditions employing headphones or other ear-level hearing devices.

## Introduction

While listening with ear-level devices, such as headphones, earphones, or hearing aids, it is often reasonable to assume that the presented acoustic signal is perceived with the same loudness as when presented *via* a loudspeaker, if the same acoustic signal is produced at the subject’s eardrum at the same sound pressure level in both conditions. This “matching assumption” is important, e.g., for free-field equalization of headphones, for virtual reality applications, for hearing device fitting, or for protecting the earphone user from hazardous high sound pressure levels (Munson and Wiener, 1952; Killion, 1978; Rudmose, 1982; Fastl et al., 1985; Keidser et al., 2000). However, there is considerable evidence in the literature (see below) about a mismatch between headphone and loudspeaker presentation violating the “matching assumption” for yet unclear reasons. This contrasts with findings from more recent research (Völk and Fastl, 2011; Brinkmann et al., 2017) indicating that virtually no mismatch occurs if the individual sound filtering properties are adequately taken into account (i.e., using individual head related transfer functions, HRTFs, and headphone related transfer functions, HpTFs), thus ensuring that the same waveforms are created at the eardrums in both presentation modes. However, the reason why these studies provide contradicting findings and how the mismatch between headphone and loudspeaker listening might depend on the different experimental parameters employed in the various studies in the literature is yet unclear. The current study therefore attempts to pinpoint the origin of the mismatch by systematically investigating the influence of room acoustics, binaural parameters, and the stimulus on the reported mismatch, as well as potential lab-specific effects.

Beranek (1949) already reported that headphones require a 6–10 dB higher level at the eardrums to provide the same loudness impression as a loudspeaker in free field. This was confirmed by Munson and Wiener (1952) who reported a “6 dB mismatch” at low frequencies for diotic headphone presentation, which they explained by different perceived positions of the source. Further confirmation of the “missing 6 dB” was reported by Robinson and Dadson (1956) and Theile (1986). Rudmose (1982), however, reported to have resolved the “case of the missing 6 dB” by attributing its existence to transducer distortions, and the procedures employed including appropriate training of the subjects and structure-borne sound transmission from the electroacoustic transducers to the subject’s body. The positioning of the loudspeaker was also acknowledged as an important factor, which was confirmed by Keidser et al. (2000) who found a mismatch of 8 dB for sounds around 500 Hz and no such difference around 3 kHz.

The observations outlined above were made under anechoic conditions, with diotic headphone presentation and a direct comparison between headphone and loudspeaker presentation, where the headphone was put on and off by the subject. Contrary, experimental designs using individual dynamic binaural synthesis, where headphones remained in place during loudspeaker playback, such that the subject was not informed which source they were listening to, achieve an authentic headphone presentation where no mismatch appeared (Völk and Fastl, 2011; Brinkmann et al., 2017). In a similarly blinded comparison, Bonnet et al. (2018) reported that an occlusion of the ear during stimulation by an external sound source did not result in a loudness mismatch to stimulation of the unoccluded ear with the same external sound source. Very recently, Meunier et al. (2020) compared loudness growth functions for headphone and loudspeaker presentation without a direct comparison of both sources, and also found no loudness mismatch. None of the experiments summarized above focused on the role of binaural hearing and interaural disparity for the mismatch. Their possible importance for the mismatch is highlighted by experiments performed by Edmonds and Culling (2009) who found a distinct influence of the interaural coherence (IC) of headphone stimuli in loudness judgment. Also, findings from Rudmose (1982) and Zahorik and Wightman (2001) using stimuli with varying distance of loudspeaker indicate that the differences in interaural coherence or the reverberant sound field influence loudness judgments. Hence, the binaural listening mode and the interaural coherence – which is usually also connected to the apparent source width (Rudmose, 1982; Zahorik and Wightman, 2001; Sivonen and Ellermeier, 2006) – appears to play an important role in the differential judgment of loudspeaker vs. headphone presentation. However, the specific influence of binaural reproduction parameters or the room on the perceived mismatch between headphone and loudspeaker presentation has not yet been assessed in a systematic way.

Another factor that might play a role in the reported loudness mismatch and the inconsistent study results is the interindividual variability in loudness perception. It is of considerable size if binaural hearing and binaural summation of loudness comes into play: Oetting et al. (2016) reported individual differences in categorical loudness scaling for the combined effect of loudness summation across both ears and across frequency that ranged up to 20 dB in effect size. Even though it is still unclear how to model these effects in current loudness models (e.g., Pieper et al., 2016), this high interindividual variability in binaural loudness summation might contribute to interindividual variability in the loudness mismatch between headphone and loudspeaker stimulation when broadband signals and an altered interaural coherence is involved.

The aim of the current study therefore is to systemically investigate the influence of a number of relevant parameters on the apparent mismatch in order to pinpoint its origin and the reason for non-consistent findings in the literature. Moreover, a thorough understanding of the influence of different parameters on the mismatch between headphone and loudspeaker presentation should be useful for avoiding this mismatch in designing modern ear-level communication systems such as, e.g., hearables or assistive listening devices. This paper focuses on the effect of room acoustics and interaural coherence on the mismatch while open-back headphones are used. Note that the influence of different kind of headphones on the mismatch is beyond the scope of the current study and will be examined in a companion paper by Kohnen et al. (in preparation).^1^

The study was designed to address the following hypotheses that are based on possible explanations for the differences across studies reported above:

- *H1: The same results with respect to the mismatch should be achieved across different labs if the same set of subjects and comparable conditions are used*. For testing this hypothesis, we performed a comparative study across two sites [Aachen (AC) and Oldenburg (OL)], employing the respective large anechoic room at each site and a group of subjects that performed the same experiments at both sites in addition to separate subjects at both sites. We extended this comparison across sites by including one additional moderately reverberant room at each site (termed as “non-anechoic” in the following, see below).
- *H2: The binaural presentation mode (diotic versus binaural headphone playback with different values of the interaural coherence) has a significant influence on the mismatch*. Hence, we used monaural as well as bilateral headphone presentation, the latter with diotic or dichotic playback. To systematically vary the reverberation time and, hence, the effective IC in the non-anechoic room as well as binaural headphone presentation, we performed the loudness matching experiments in four different rooms: The anechoic rooms in Oldenburg and Aachen, a sound-insulated lab room with little reverberation (OL earpiecelab, T30 = 0.4 s) and a medium-sized room without any specific acoustical treatment (AC tea kitchen) exhibiting a reverberation time T30 of approx. 0.6 s (see Table 1).
- *H2a: No mismatch between headphone and loudspeaker presentation in a non-anechoic room can be observed if the interaural coherence during headphone presentation is matched to the respective room*. To test this hypothesis, the “IC matched” condition was additionally tested throughout the experiments listed above.
- *H2b: The apparent source width is strongly connected to the mismatch*. To test this hypothesis, the apparent source width in the different experimental headphone playback conditions in comparison to the apparent source width of the target loudspeaker was evaluated and compared to the mismatch results.
- *H3: The interindividual spread in the mismatch across different conditions is related to the individual variability in binaural loudness summation or other individual binaural processing characteristics (like, e.g., the binaural benefit in a spatial speech recognition task)*. To test this hypothesis, we performed additional audiological evaluations with a subset of the subjects employed here.

## Materials and Methods

Subjects matched the perceived loudness of a headphone presentation to that of a loudspeaker presentation of the same stimulus, and the levels at the eardrum for equal loudness were compared. No equalization of the loudspeaker or headphone was applied during stimulus presentation. The loudness matching experiment was performed in four rooms distributed over two sites, three headphones, for four signals, and four headphone presentation modes (section Stimuli and Rooms). In this paper, only the results obtained with open-coupling headphones (HD 650, Sennheiser, Wedemark, Germany) are presented. The HD650 was chosen here due to its widespread use and due to low repositioning variation compared to the other headphones tested (Beyerdynamic DT770 Pro, Etymotic ER4, for further details see^1^), which does not depend on a tight fit on the ear due to the open-back design. Subjects underwent four experimental sessions at each site, including one session for auditory screening and characterization (section Subjects and Characterization), one for measurements of individual ear-related transfer functions (section Sound Levels at Eardrum), and two for the loudness matching and apparent source width experiments (section Procedure and Apparatus) that were separated between the two room conditions. All possible conditions in each room (Stimulus x Headphone Presentation Mode) were performed in random order. A part of the subjects conducted the experiments at both sites to assess possible lab-specific effects and reveal potential errors more easily. Table 1 shows a summary of all conditions.

**Table 1:** Keys and description for each condition.

Room	**OL_anechoic** Oldenburg Virtual Reality lab	**AC_anechoic** Aachen hemianechoic chamber	**OL_earpiecelab** Oldenburg shoebox-shaped sound isolated lab room T_30_ = 0.395 s	**AC_teakitchen** Aachen, non-shoebox shaped room, former tea kitchen T_30_ = 0.574 s
Signal	**tbn250** Third-octave-band noise, center frequency 250 Hz	**tbn1000** Third-octave-band noise, center frequency 1,000 Hz	**tbn4000** Third-octave-band noise, center frequency 4,000 Hz	**uen17** Broadband Unified Excitation Noise, same energy in 17 auditory filters between 20 Hz and 4 kHz
Headphone Presentation Mode	**Monaural** Presentation on left ear only	**Diotic** Same sound on both ears	**IC matched** Interaural coherence matched to room	**Uncorrelated** Independent sound samples at both ears

Each row shows the possibilities of the factor denoted in the left column. See main text for more details.

### Procedure and Apparatus

The loudness matching experiment was implemented as a 1-up-1-down alternative forced choice paradigm (Levitt, 1971; Kollmeier et al., 1988) implemented in the AFC toolbox (Ewert, 2013). At all times, the subjects were aware whether the sound was presented from the headphones or the loudspeaker, and they saw their surroundings including the loudspeaker. For each condition, the loudness matching experiment began with loudspeaker presentation of the stimulus. The subjects then put on the headphones and started the headphone presentation by pressing a button on a foot switch. They then indicated whether the presentation on the headphone or the loudspeaker was perceived as louder, and the headphone playback level was adapted accordingly. A foot switch with three buttons (“Continue,” “Headphone was Louder,” and “Loudspeaker was Louder”) allowed the subjects to quick response to instructions presented on a screen positioned on the floor in front of them, while they had the hands free for handling the headphones. The presentation order was alternated between trials, such that repositioning of the headphone was reduced to a minimum. The stepsize of the headphone playback level was reduced from the initial 10 to 5 dB and 1 dB after the first and second upper reversals, respectively. The initial headphone playback level was always chosen such that the loudspeaker was perceived as louder, which – in combination with the large initial stepsize – worked as a “bracketing” of the assumed level of equal loudness, thus reducing any bias produced by the selection of the start level. The median value of the three upper and lower reversals of the headphone playback level during the measurement phase was stored as the resulting equal-loudness level. A typical matching process needed between 10 and 20 comparisons until convergence was reached, which took approx. 1–2 min for each condition and approx. 60–80 min for a full session. Pauses were allowed after each condition and after one-third and two-thirds of the whole experiments were completed. Frozen stimuli were used, i.e., the same waveforms (except level adjustments for the headphone) were presented on each iteration. Sound pressure levels at the eardrum with loudspeaker and headphone presentation at equal loudness were calculated *post hoc* using individually measured transfer functions as described in section Sound Levels at Eardrum.

The loudspeaker was a Genelec 8030 active studio monitor that was mounted in view direction and head height (1.25 m) of the seated subjects at 2.25 m distance. The subjects were instructed to point their heads toward the loudspeaker at least during loudspeaker presentation. The loudspeaker presentation level was set to 65 Phon as per (ISO 226, 2003) for a pure tone at the center frequency of each stimulus (see section Stimuli and Rooms, 1 kHz for the broadband stimulus) to present all stimuli at roughly similar loudness. The loudspeaker presentation level was calibrated using a ½” free-field microphone (46AF, G.R.A.S., Holte, Denmark) pointed at the loudspeaker and mounted at the position of the subject’s head. Both the loudspeaker and the headphone were connected to a laptop using an ADI-2 Pro FS sound interface (RME, Haimhausen, Germany) through its line and high-power headphone outputs, respectively.

Also, an experiment assessing the apparent source width of the headphone presentation with respect to the loudspeaker presentation was conducted. To this end, we adapted a graphical user interface originally designed for sound quality assessment (Völker et al., 2018). The interface was shown on a touch screen and consisted of a rating panel with a horizontal scale for the apparent source width ratings and buttons representing the different conditions. The loudspeaker playback served as the reference and could be started by pressing the appropriate button, which was fixed at the center of the panel. Pressing of the three other buttons started playback of the same stimulus over headphones with different interaural coherence (see section Stimuli and Rooms) at levels that were previously determined as equally loud as the loudspeaker playback. Monaural headphone presentation was not included in this experiment. The buttons could be positioned in the panel *via* drag and drop to indicate the apparent source width as compared to the loudspeaker presentation. The panel was labeled with a numerical scale ranging from −50 to 50, supplemented by descriptions (much smaller, smaller, larger, and much larger positioned at −40, −20, 20, and 40, respectively). Thus, negative values here indicate a smaller, 0 an equal, and positive values indicate a larger apparent source width in headphone presentation. A separate run of the interface was started for each of the four signals (see section Stimuli and Rooms). The experiment was only conducted in the non-anechoic rooms, in the same session, and directly after the loudness matching experiments were finished and lasted another approx. 10 min.

### Stimuli and Rooms

Four different signals were used. Three signals were one-third-octave-band noises with center frequencies at 250 Hz, 1 kHz, and 4 kHz (referred to as tbn250, tbn1000, and tbn4000 in the following). The fourth was a broadband noise with equal energy in each of 17 critical frequency bands as defined by Zwicker (1961) in a frequency range between approx. 250 and 4 kHz, i.e., the same lower and upper boundary frequency as the narrowband noises. The signals were chosen to capture frequency regions with a high (250 Hz), intermediate (1 kHz), and low (4 kHz) ability of the human auditory system to integrate the temporal fine structure across the two ears (Moore, 2012). Also, differences between narrow-band sounds that fall within one auditory filter and broadband sounds can be characterized. In contrast to many other studies on loudness, the temporal envelope of the one-third-octave-band stimuli was not flattened (Kohlrausch et al., 1997) to facilitate manipulations of the interaural coherence in headphone presentation. The signals were 1 s in duration including 20 ms long rise and fall ramps. All level calculations excluded the ramps and possible reverberant tails.

Four different headphone presentation modes were employed for the headphone presentation:

- Monaural: presentation on left ear only.
- Diotic: same signal presented on both ears, interaural coherence = 1.
- IC matched: Interaural coherence matched to loudspeaker presentation.
- Uncorrelated: Independent noise samples presented on both ears, interaural coherence = 0.

**Figure 1:**
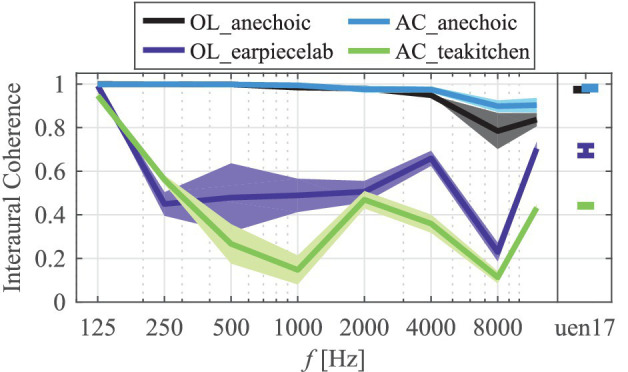
Measured interaural coherence values of the rooms, measured in third octave bands, and for the broadband uen17 stimulus using a KEMAR. Thick lines incdicate average values and shaded areas indicate standard deviations across several positions around the reference head position.

Binaural stimuli with arbitrary interaural coherence were created by adding two independent noise samples with appropriate weights (symmetric generator method, Hartmann and Cho, 2011). The signal presented on the loudspeaker was always identical to the signal presented to the left ear over the headphone. The interaural coherence with loudspeaker presentation was determined using a KEMAR 45BB-12 mannequin with anthropometric pinnae and low-noise ear simulators (G.R.A.S., Holte, Denmark). The interaural coherences for third-octave band and the uen17 stimuli and rooms including observed standard deviations across several positions in a 20 cm radius around the reference position of the head are shown in Figure 1.

The experiments were conducted in an anechoic chamber and one office-like non-anechoic room at both sites in Oldenburg (OL) and Aachen (AC). At Oldenburg, a full anechoic chamber sized 8.6 m × 5.8 m × 5.5 m with 0.6 m foam wedge absorbers and a setup of 94 loudspeakers was used (OL_anechoic). While the loudspeakers generate mild reflections in the mid frequency range (Denk et al., 2018b), the reverberation time is still below 60 ms above 100 Hz. The non-anechoic room in Oldenburg was an isolated lab within a room with a shoe box shape (5.15 m × 3.85 m × 3.5 m) and a T_30_ reverberation time of 0.395 s (OL_earpiecelab). At Aachen, a hemianechoic chamber with a rigid floor of size 11 m × 5.97 m × 4.5 m and 0.8 m wedge length was used (AC_anechoic). The reflection from the floor was additionally attenuated through a 0.5-m foam wedge absorber layer laid out on the floor between the subject and the loudspeaker. The non-anechoic room in Aachen is the institute’s old tea kitchen, which is non-shoebox (higher ceiling at approx. 1/3 of the ground area) with a ground area of approx. 2.7 m × 5 m, and average height of approx. 3 m, and a T_30_ reverberation time of 0.540 s (AC_teakitchen). In both non-anechoic rooms, the subjects and the loudspeaker were positioned asymmetrically to decorrelate the signals at both ears. The distance from the loudspeaker was at least a factor of four larger than the reverberation radii (OL_earpiecelab: 0.5 m, AC_teakitchen: 0.32 m, using Sabine’s formula), i.e., the level of the reverberant sound field dominates at the position of the subjects. Room acoustic parameters were determined using the loudspeaker used in the loudness matching experiments and a free-field microphone (46AF, G.R.A.S., Holte, Denmark) positioned at the location of the subjects’ head.

In the OL anechoic chamber, one of the installed Genelec 8030 loudspeakers was connected to the experimental laptop. This loudspeaker was mounted on a traverse system that was ultimately mounted on the supporting steel beam structure at the ceiling of the chamber. In all other rooms, the loudspeaker was mounted on a microphone stand on the floor.

### Subjects and Characterization

Forty normal-hearing subjects (27.6 ± 7.2 years of age, half male and female, including three authors) participated in the study. Fourteen subjects (gender-balanced) went through the measurements at both sites, and additional 13 subjects were measured at each site, amounting to a total of 27 subjects measured at each site. The 14 subjects that went through the identical experiments at both sites allowed for a direct comparison of results and served to reveal any lab-specific differences.

Pure-tone audiometry with extended high frequencies was performed using an automated method (Bisitz and Silzle, 2011) to verify that the subjects had normal hearing. Subjects were excluded if their threshold exceeded 20 dB HL at one single audiometric frequency up to 8 kHz, or 35 dB HL at 12.5 or 16 kHz. For subjects participating in Oldenburg, further auditory characterization was conducted. This included the assessment of monaural and binaural loudness growth functions for the stimuli of the present study using the adaptive categorical loudness scaling method (ACALOS; Brand and Hohmann, 2002). Note that in the loundess growth function experiment, the narrowband stimuli had an optimized temporal envelope with minimal temporal level variations but the same spectrum (“low-noise noise”; Kohlrausch et al., 1997). Also, the SNR at 50% speech intelligibility (SRT50) was determined for a frontal speech source and noise at the front or the right, both with the left ear only and binaurally using the Oldenburg sentence test (Wagener et al., 1999). The subjects conducted all measurements autonomously using the Oldenburger Measurement Applications (Hoertech, 2019) with appropriate GUIs and using HDA300 audiometric headphones (Sennheiser, Wedemark, Germany).

### Sound Levels at Eardrum

The sound pressure levels at the eardrum of the subjects were calculated *post hoc* using individually measured transfer functions. That is, the levels during headphone presentation were calculated by convolving the headphone stimulus (voltage at a level that produced equal loudness as free-field presentation) with individual HpTFs. The levels at eardrum during loudspeaker presentation were computed by convolving the loudspeaker stimulus (pressure waveforms at free field, known by calibration) with individual HRTFs. The transfer function-based calculation has the benefit that the same transfer function can be used for multiple conditions and sessions. Also, in transfer functions, it is easier to recognize faulty measurements (e.g., spurious notches due to placement too far away from the eardrum) and eliminates those from further calculations than in direct measurements of narrow-band sound pressures at the eardrum. We verified the transfer function-based approach against direct measurements of the stimuli in all rooms using the KEMAR.

The transfer functions to the eardrum were measured using probe tube microphones (ER7C, Etymotic Research, Elk Grove Village, IL, United States). The probe tubes were inserted into the ear canal until the subject reported contact with the eardrum, and then pulled back by a minimal amount and fixed at the check using medical tape. Comparatively, long probe tubes of 76 mm length (Type 76109MBB, Precision Cast Plastic Parts, Redding, CA, United States) were used, such that it was possible to place the body of the probe microphone outside of the headphone cushion to avoid leaks. Transfer function measurements were conducted in the anechoic chambers at each site. The transfer functions of the 14 subjects participating at both sites were measured at both sites, and for level calculations the transfer functions measured at the site of the appropriate room were utilized.

**Figure 2:**
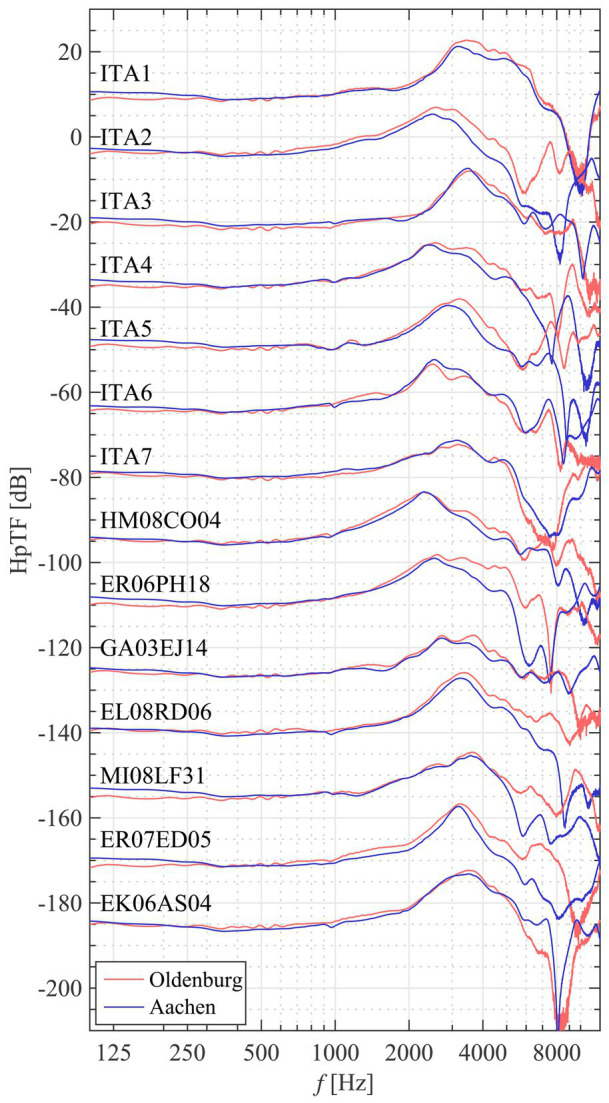
HpTFs (power spectrum averages across eight repetitions, right ear) of the subjects that went through measurements at both sites. A good correspondence below 5 kHz verifies the validity of measurements at both sites in the frequency range of interest. Individual curves have been shifted in increments of −15 dB with respect to the top one for better display.

The HpTF was measured eight times using exponential sweeps including repositioning of the headphone to account for known variabilities (Kulkarni and Colburn, 2000; Müller and Massarani, 2001). In the frequency range of interest here (0.25–4 kHz), the typical standard deviation lies around 3 dB between subjects and 1 dB within one subject. The within-subject variations are in the same range as reported by Völk (2014) for 50 repetitions, showing that the eight repetitions employed here are sufficient to capture the variations that also occur during the listening tests when the subjects put the headphones off and on. The stored stimulus waveform that was presented during the psychoacoustic experiment was convolved with each instance of the HpTF, the RMS calculated for each ear and HpTF instance separately, the RMS values averaged and then transformed to dB SPL. The random variations of the HpTF included in the listening test, which contribute to the overall uncertainty of the results, are thus included in the level calculation procedure. For the monaural presentation mode, only the ear, where sound was presented, was regarded. HpTFs measured at both sites for the 14 cross-site subjects are shown in Figure 2, and a good correspondence between sites especially up to 4 kHz demonstrates a high data quality. Note that different headphones bought in one batch were used at both sites.

**Figure 3:**
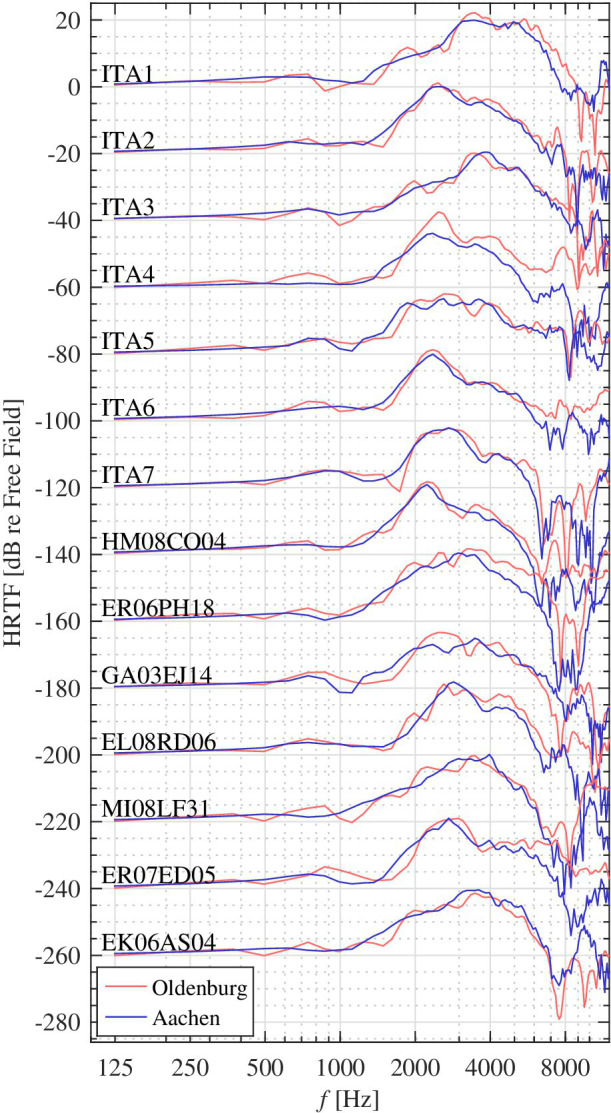
HRTFs for frontal incidence in the left ear of the subjects that went through measurements at both sites. A good correspondence below 8 kHz verifies the validity of measurements at both sites in the frequency range of interest. Individual curves have been shifted in increments of −20 dB with respect to the top one for better display.

The transformation from free field to the eardrum of the subject for a specific incidence direction is defined by the HRTF. HRTFs were measured for each subject in 87 and 3072 directions in Oldenburg and Aachen, respectively, using the techniques described in Denk et al. (2018a) and Richter (2019) in the same session as the HpTFs without repositioning of the probe tube. HRTFs for frontal incidence of the subjects that went through measurements at both sites are shown in Figure 3, and again a good correspondence demonstrates a generally high data quality. One subject participating only in Aachen had to be excluded due to a faulty HRTF measurement that could not be repeated due to the Corona pandemic.

**Figure 4:**
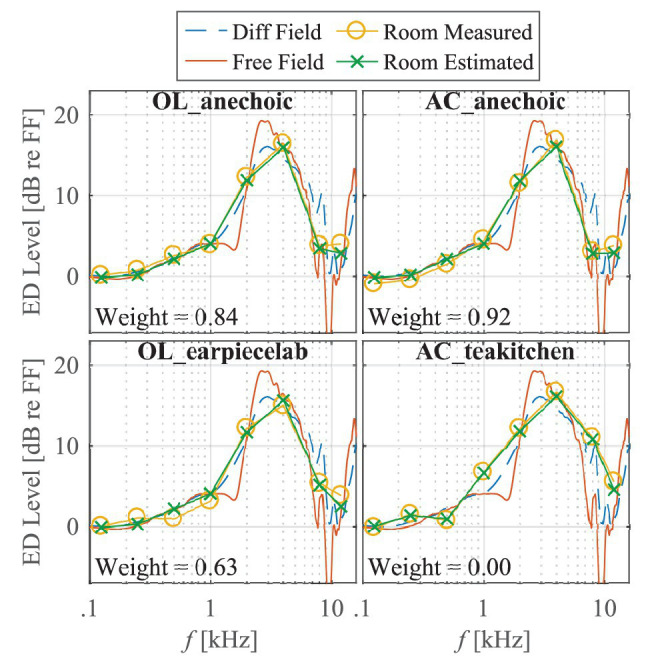
Third-octave noise levels at eardrum (ED) with respect to free field measured in KEMAR (circles). Free‐ and diffuse-field responses are shown as solid red and dashed blue lines, respectively; estimated levels are free field obtained from KEMAR HRTFs and room-specific weighting factors are depicted as green crosses.

For loudspeaker presentation, the level at free field at the location of the subject’s head is known by calibration. In case of the anechoic chambers, a stimulus at eardrum and its corresponding level can thus be calculated by convolving the loudspeaker stimulus with the HRTF for frontal incidence. In the non-anechoic rooms, sound is reflected from the walls, the ceiling, and the floor, such that the sound field includes incidence from many other than the frontal incidence direction. This room-specific effect was approximated by a weighted average of the magnitudes of individual HRTFs for free‐ and diffuse-field incidences, representing the direct sound from the loudspeaker and the diffuse room reverberation. The individual diffuse-field HRTF was approximated by power spectrum averaging a subset of HRTFs uniformly distributed in space (Denk et al., 2018a). The weight between free‐ and diffuse-field incidence was adapted to each room (including anechoic chambers) to match KEMAR measurements of the stimuli level at eardrum in this room. This weight was used for each subject to compute a “room-matched HRTF” from individual free-and diffuse field HRTFs. This simple model matched the measured data with an accuracy of ±1 dB for the frequencies of interest, except for the AC_teakitchen. In this room, a prominent early reflection limited the accuracy of this model. The room-matched HRTF for this room was thus extended by an additional heuristic correction, which comprised the difference between estimated and measured levels in KEMAR.^2^ The measured levels in KEMAR together with KEMAR’s frontal‐ and diffuse-field HRTF and the weighted average are shown in Figure 4.

## Results

### Level Mismatch at Equal Loudness

**Figure 5:**
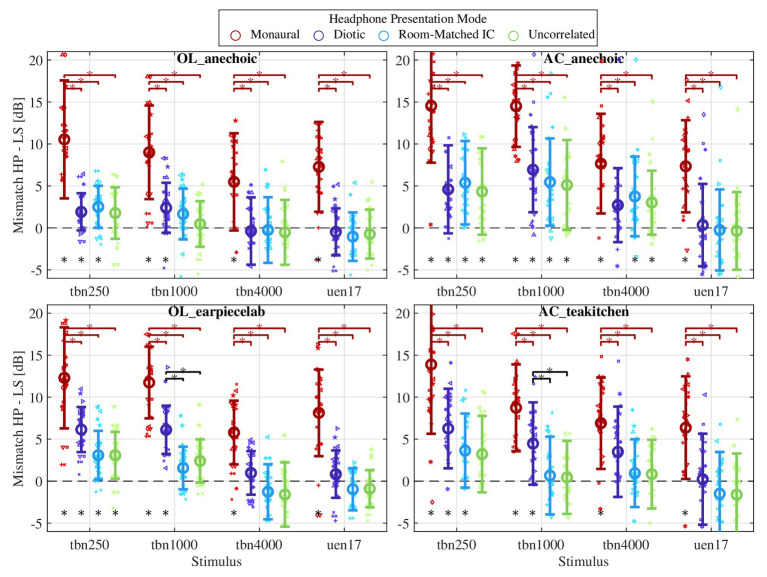
Mismatch of levels at eardrum at equal loudness, headphone level minus loudspeaker level. Results for each room are shown in individual panels, the position on the *x*-axis denotes the stimulus, and the color denotes the Headphone Presentation Mode. Small symbols denote individual subjects, large symbols denote the mean, and error bars denote the standard deviation across subjects for each condition. A star at the bottom denotes a statistically significant mismatch for this condition, and stars above brackets indicate a significantly different mismatch between conditions connected by the bracket.

Figure 5 shows the observed mismatch (headphone level minus loudspeaker level at eardrum at equal loudness in each subject) separately for each room, stimulus, and headphone presentation mode. For each condition, i.e., the combination of room, stimulus, and headphone presentation mode, the statistical significance of the difference from a mean of zero was assessed by *t*-tests including a Bonferroni correction for 64 paired comparisons. Statistically significant differences (*p* < 0.05) are marked by stars below the appropriate error bar in Figure 5. Aside from the conditions with monaural headphone presentation that are further assessed in section Binaural Parameters and Level Mismatch, a significant mismatch in the range of 3–6 dB is generally observed for the tbn250 stimulus. For the tbn1000, a significant mismatch is noted in all rooms with diotic headphone presentation, and with all headphone presentation modes in the AC anechoic chamber. For the tbn4000 stimulus, a significant mismatch is only observed in the AC anechoic chamber and room-matched and uncorrelated headphone presentation, although a trend towards a mismatch is also visible for this stimulus and diotic headphone presentation in both AC rooms. For the broadband uen17 stimulus and either binaural headphone presentation mode, no mismatch is observed.

Factors influencing the mismatch were further analyzed by means of a three-way ANOVA with the factors Room, Stimulus, and Headphone Presentation Mode.^3^ Significant effects were revealed for all factors [Room: *F*(3, 1,630) = 25.1), *p* < 0.001; Stimulus: F(3, 1,630) = 103.0), *p* < 0.001; Headphone Presentation Mode: F(3, 1,630) = 322.9, *p* < 0.001], as well as all possible 2-way interactions [Stimulus × Headphone Presentation Mode: *F*(9, 1,630) = 2.6, *p* < 0.001; Stimulus × Room: F(3, 1,630) = 6.3, *p* < 0.001; Room × Headphone Presentation Mode: F(3, 1,630) = 2.7, *p* = 0.003]. The three-way interaction term was not significant [*F*(27, 1,630) = 0.358, *p* = 0.99].

**Figure 6:**
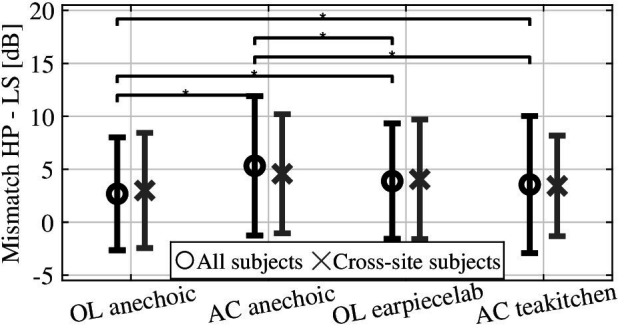
Marginal means of mismatch observed in all rooms, mean, and standard deviation shown for all subjects (black symbols), and the subset of subjects that participated at both sites (cross-site subjects, gray symbols).

As revealed by the ANOVA explicitly visible in the marginal means of the rooms as shown in Figure 6, the mismatch differs between rooms. These differences were assessed by means of a *post hoc* test on the marginal distributions for all subjects including a Bonferroni correction for six paired comparisons. An appropriate evaluation of the cross-site subjects’ data that is shown for comparison in Figure 6 yielded equivalent statistical results. On the one hand, significant differences between both anechoic chambers [∆ = 2.62 ± 0.30 dB (mean difference ± standard error), *p* < 0.001] with higher mismatch values in the Aachen chamber are noted. On the other hand, no significant difference is seen between the non-anechoic rooms at both locations (Δ = −0.33 ± 0.31 dB, *p* = 1). At Oldenburg, a larger mismatch is seen in the non-anechoic rooms than in the anechoic chamber (OL: Δ = 1.12 ± 0.30 dB, *p* = 0.001), while in Aachen the mismatch values are larger in the anchoic chamber (Δ = 1.76 ± 0.31 dB, *p* < 0.001). The mismatch was generally larger in the AC anechoic chamber as compared to the OL non-anechoic room (Δ = 1.42 ± 0.31 dB, *p* < 0.001), while the mismatch was smaller in the OL anechoic room as compared to the Aachen non-anechoic room (Δ = −0.86 ± 0.31 dB, *p* = 0.03).

Differences in the mismatch between stimuli are rather consistent between Headphone Presentation Modes in each room but differ between rooms. In both Oldenburg labs, the observed mismatch is very similar between the tbn250 and tbn1000, and larger in these two stimuli than with the tbn4000 or broadband uen17, where no mismatch is evident (except for monaural headphone presentation). In the AC_anechoic chamber, mismatches were slightly larger with the tbn1000 stimulus than with the others, while in the AC_teakitchen, only a minor dependence on the stimulus is seen. Common to all rooms is that no mismatch is observed with the broadband uen17 stimulus presented binaurally. Significant differences between marginal means of the stimuli were observed in all possible comparisons.

Differences between Headphone Presentation Modes were assessed within each combination of Stimulus and Room (as grouped in Figure 5, stars above bracket between conditions indicates *p* < 0.05) by pairwise *t*-tests with Bonferroni correction. First, little surprisingly there is a significant difference between monaural vs. all binaural headphone presentation modes. Second, in the non-anechoic rooms (OL_earpiecelab and AC_teakitchen), there is a tendency that the mismatch is larger in diotic vs. room-matched or uncorrelated headphone presentation, irrespective of the stimulus. However, this trend only reaches significance for the tbn1000 stimulus. This influence of the interaural coherence is exclusively seen in the non-anechoic rooms, i.e., where the interaural coherence is also considerably different from 1 with loudspeaker presentation (cf. Figure 1). Third, no considerable trends or significant differences are seen between uncorrelated and room-matched headphone presentation. Further evaluations regarding the influence of interaural coherence of the headphone presentation is given in section Binaural Parameters and Level Mismatch.

### Binaural Parameters and Level Mismatch

**Figure 7:**
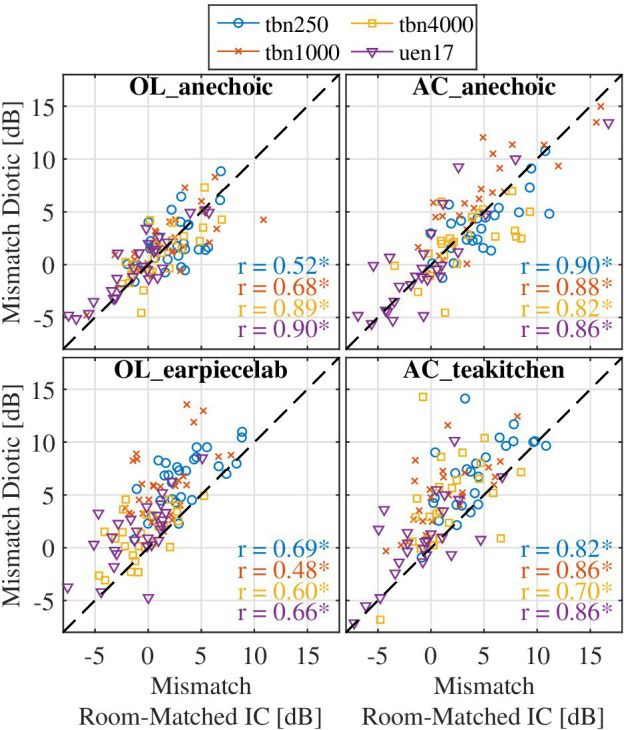
Individual mismatch to loudspeaker presentation with room-matched (*x*-axis) or diotic (*y*-axis) headphone presentation. Each symbol denotes the result for one subject, and different stimuli are indicated by colors and symbols. The Pearson correlation coefficient for each stimulus is given by the values in the lower right corner of each panel, and a star behind the value denotes a statistically significant correlation.

In Figure 5, it is evident that especially in the non-anechoic rooms, a reduction of the interaural coherence in binaural headphone reproduction, on average, reduces the mismatch with respect to diotic presentation. Figure 7 shows the individual correspondence of the mismatch with diotic and room-matched IC headphone presentation, separated for the different rooms and stimuli. High and significant correlations are seen between the mismatch results with both headphone presentation modes within the subjects. In the anechoic chambers, where the IC is very close to 1 (cf. Figure 1), thus diotic and room-matched headphone presentation are very similar, the results are centered around the diagonal and highly correlated, i.e., the mismatch was repeatable. In the non-anechoic rooms (OL_earpiecelab and AC_teakitchen), the distributions have an offset to the top of the diagonal, i.e., also for the individual level, the mismatch is generally larger with diotic presentation. The high correlation shows that, while the general size of the mismatch seems to be a rather individual quantity, the reduction of mismatch by adaptation of the interaural coherence to the room seems to be a factor that is consistent across subjects.

No links of the reduction of mismatch between headphone presentation modes to individual abilities to integrate across ears were found. Correlation analysis of the mismatch differences with the benefit of adding the worse ear in a spatially separated Speech-in-Noise task or difference between monaural and diotic categorical loudness growth functions (cf. section Subjects and Characterization) did not reveal any dependences on the individual level.

The equal-loudness levels are approx. 5–9 dB larger with monaural vs. binaural headphone playback, which obviously relates to the well-known effect of binaural loudness summation (Marks, 1978; Edmonds and Culling, 2009; Oetting et al., 2016). Similar to the effect of the interaural coherence, no correlation between the difference between monaural and binaural results from Figure 5, and difference in monaural and diotic loudness growth functions was seen. However, it should be noted that the equal-loudness level difference between monaural and diotic presentation seen here is larger than the typically reported effect of binaural loudness summation in headphone experiments, which lies around 3–6 dB (Edmonds and Culling, 2009).

### Apparent Source Width and the Level Mismatch

**Figure 8:**
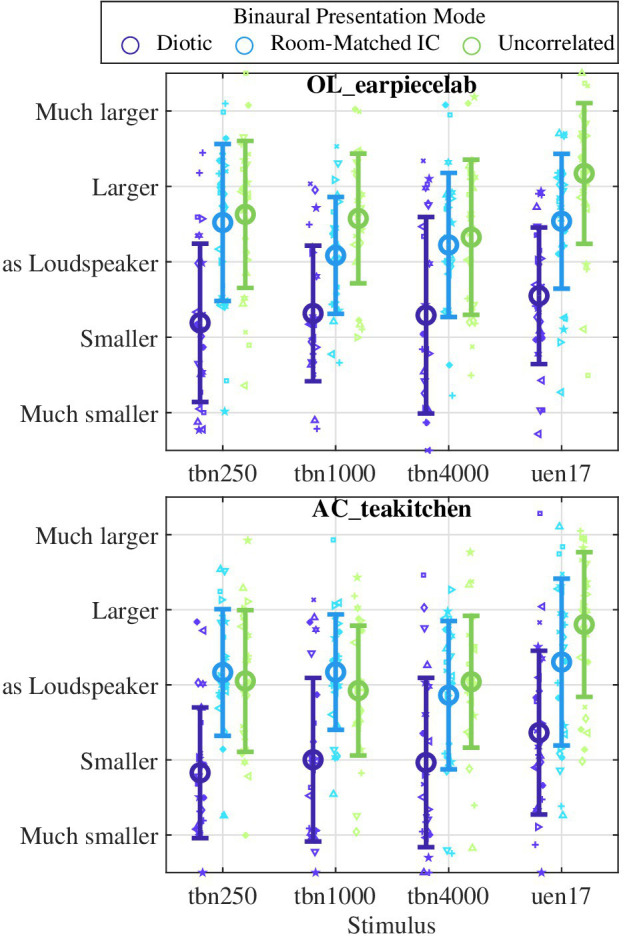
Apparent Source Width ratings for the stimuli presented over headphones with different modes (colors), separated across stimuli and the two non-anechoic rooms. Small symbols denote subjects’ ratings, and large symbols and error bars denote the average and standard deviation, respectively.

Figure 8 shows the apparent source width ratings for the headphone presentation in the two non-anechoic rooms. The ratings are very similar between rooms and stimuli. The diotic stimulus presentation was generally perceived as smaller than the loudspeaker and headphone presentation with the room-matched or zero interaural coherence. For the narrowband stimuli (tbn250, tbn100, and tbn4000), the apparent source width was rated very similarly between the room-matched and uncorrelated conditions. At the same time, the apparent source width of uncorrelated and room-matched headphone presentation was rated very similar to that of the loudspeaker in the AC_teakitchen but a bit larger than the loudspeaker in the OL_earpiecelab. Only for the broadband uen17 noise, the uncorrelated headphone playback was perceived as larger than the room-matched playback. Reduction of the interaural coherence below that of the loudspeaker presentation thus led to an apparent source width that is larger than both the loudspeaker and the room-matched headphone presentation.

The apparent source width ratings are well in line with the mismatch between loudspeaker and headphone presentation: on average, headphone stimuli that were perceived as smaller also elicited a higher mismatch (diotic vs. room-matched, cf. section Level Mismatch at Equal Loudness). However, while the influence of the interaural coherence on the mismatch is smaller at high frequencies (tbn4000) or for the broadband noise (uen17), no such dependence is seen for the apparent source width ratings. No significant correlations between individual judgments of apparent source width and the mismatch were noted, however, this may be caused by the large variance of the apparent source width judgments.

## Discussion

### Strengths and Limitations of the Current Study

The current study provides a rich dataset of loudness matching experiments with up to 40 subjects, four different rooms across two lab sites, four different binaural conditions, and four different signals that is unparalleled so far in the literature. While the investigation included three different headphone models, in the present work, only the results for the open-coupling Sennheiser HD650 are shown. Without pre-empting on the companion paper (see Footnote 1), it should be stated here that the main outcomes of the present work are no different for the other headphones.

The fact that individual HRTFs and HpTFs were recorded for each subject provided the possibility for an estimation of the mismatch in each condition that takes into account individual sound transfer characteristics of the ears both for the headphones and the loudspeaker. Contrary to the headphones and anechoic chambers, in the non-anechoic rooms, the transfer function comprises not only the measured direct transfer path between loudspeaker and eardrum, but also numerous reflections from different incidence directions and delays. This so-called binaural room transfer function was not directly measured, but modeled as a superposition of direct sound and reverberant field, where the weights of both components were determined for KEMAR and used for all subjects. While this approximation of the complex transfer behavior includes the effect of individual ear properties, it is still possible that errors in the estimated level at eardrum are introduced due to an oversimplification of the sound field. The additional heuristic correction necessary in the non-anechoic room in Aachen (cf. section Sound Levels at Eardrum), which was derived from differences between the originally estimated and measured levels for this room, gives a first estimate of the introduced accuracies. By doubling this correction, we estimate a worst-case error due to this approximation of around 3 dB. However, there is no reason why this inaccuracy should not be evenly distributed across subjects. Therefore, we assume that this estimation may lead to an increased uncertainty of the levels at eardrum for loudspeaker presentation in the non-anechoic rooms, but not to a change of the average mismatches observed.

In spite of the post-hoc compensation of individual transmission effects, sound presentation did not include any individual HpTF-compensation across frequency, but used the inherent free-field equalization of the headphones employed here. While for the three narrowband stimuli, it can be assumed that this approximation of the desired frequency response suffices to match the stimulus spectrum using headphones to that of the loudspeaker presentation, this is not the case for the broadband stimulus uen17, where coloration differences might interfere with the loudness matching task between loudspeaker and headphone presentation. However, this broadband stimulus provided the least mismatch across all conditions (cf. Figure 5), indicating that the spectral approximations during the measurement procedure do not interfere with the interpretability of the data. Nevertheless, future experiments should also perform the individual equalization of the headphones already during the measurements with broadband stimuli to test any potential influence of coloration artifacts and connected spatial cues on the loudness mismatch.

### Occurrence and Size of the Mismatch: Diotic Headphone Presentation

With diotic headphone presentation, a significant mismatch of 3–7 dB higher level at eardrum with headphone as compared to loudspeaker presentation was consistently seen for narrow-band sounds at frequencies lower than 4 kHz. The mismatch occurred both in anechoic and non-anechoic conditions and was in each room very similar in size for the stimuli at 250 and 1,000 Hz. Our data hence confirm previous studies, e.g., Munson and Wiener (1952) and Keidser et al. (2000), indicating that for low frequencies up to 1 kHz a significant mismatch exists, albeit slightly smaller than the 6–8 dB reported previously. For the 4 kHz stimulus, no significant mismatch was observed in either room, although there is a tendency toward a mismatch of approx. 3 dB in both AC rooms, which is discussed below. For broadband stimuli, our results show very clearly that there is no mismatch, specifically confirming results by Brinkmann et al. (2017) who used binaural synthesis instead of diotic headphone playback.

For diotic headphone presentation and frequencies below 4 kHz, the occurring mismatch is smaller in the OL anechoic lab as compared to the other rooms (approx. 3 vs. 6 dB). While at OL, the mismatch is larger in the non-anechoic room, at AC, the mismatch values were similar in anechoic and non-anechoic conditions. Between the anechoic chambers at both sites, we see a striking and statistically significant difference of approx. 3 dB for all narrowband stimuli (incl. 4 kHz) and diotic headphone playback. These differences are also significant for our subset of 14 subjects who performed the experiment at both labs. Faulty calibration of equipment as a source of the difference between sites can be mostly ruled out due to the consistently non-existent mismatch with the broadband stimulus, and given the good correspondence between sites in the non-anechoic rooms. However, small differences in the experimental setup were unavoidable between the anechoic rooms in Oldenburg and Aachen (cf. section Stimuli and Rooms). Room acoustical consequences of these small differences included a smaller interaural coherence in the OL anechoic room (Figure 1), and a potential floor reflection in the AC anechoic room despite laying out absorbers on the floor. Vibration (Rudmose, 1982) might have a lower influence in the OL anechoic chamber due to the loudspeaker mounting on traverse system as opposed to a stand on the floor in the other rooms, although we do not expect vibration to reach a significant level in general. It should be stressed that all these acoustic factors would cause frequency-specific effects, while the observed difference in mismatch is very consistent across narrowband stimuli. Another possible explanation for the difference between anechoic rooms are non-acoustic differences such as the general impression of the room and visual cues like seeing one vs. many loudspeakers. While the potential influence of visual cues on loudness judgments is well-known, the presence of a total of 94 spatially separated loudspeakers in the OL anechoic lab is a feature of the experimental room that cannot be easily changed. The influence of such non-acoustic factors on the loudness mismatch should therefore be assessed in future experiments, e.g., by blindfolding subjects of providing different visual cues on a head-mounted display.

Contrary to the anechoic rooms, the mismatch results between the two non-anechoic rooms are quite consistent between both sites. These rooms were visually rather similar (single loudspeaker mounted in empty room) but acoustically different (T30 = 0.4 s vs. 0.57 s), although one may argue that the perceived difference between these rooms may be smaller than deviations from anechoic properties in one of the anechoic chambers. Altogether, we discard our hypothesis H1 and must conclude that small differences in the setup of the especially anechoic test conditions may lead to a considerable difference in obtained mismatch between diotic headphone and loudspeaker presentation. This might also explain the inconsistent reports from the literature about the (non-) observation of this mismatch since the experiments were all performed in somewhat different room conditions (Munson and Wiener, 1952; Rudmose, 1982; Fastl et al., 1985; Völk et al., 2011; Bonnet et al., 2018).

### Influence of the Headphone Presentation Mode and Apparent Source Width

The interaural coherence in headphone presentation (diotic vs. room-matched/uncorrelated IC) exhibits a significant influence on the obtained loudness mismatch in the non-anechoic rooms, but virtually no difference in the anechoic chambers (cf. Figure 5). Also, no difference is evident between room-matched and uncorrelated headphone presentation. In the present data, the trend toward a difference in mismatch between diotic and room-matched/uncorrelated presentation in the non-anechoic rooms amounts up to 5 dB and is visible for all stimuli including the broadband sound. However, it only reaches significance for the 1,000 Hz narrowband stimulus (cf. Figure 5). The trend to smaller mismatches with uncorrelated headphone presentation in the non-anechoic rooms is consistent with results of Edmonds and Culling (2009), who reported lower levels in uncorrelated vs. diotic headphone presentation at equal loudness. The effect in their data was slightly smaller (up to 3 dB in size) and declined toward high frequencies and large bandwidth similarly to our data. However, given their data, it is quite surprising that the interaural coherence of the headphone presentation does not influence the mismatch to the loudspeaker – if the diotic/room-matched headphone playback (IC≈1 in the anechoic chambers) would have been directly compared with uncorrelated playback, a lower level at equal loudness would have been expected with the uncorrelated presentation. In conclusions, hypothesis H2 (influence of binaural presentation mode) can be supported for non-anechoic, but not for anechoic environments. Hypothesis H2a (matching the IC eliminates mismatch) has to be rejected: A mismatch was still significant with room-matched interaural coherence in all conditions where it was significant with diotic presentation, albeit reduced in non-anechoic conditions.

A closer look into the individual variations of mismatch in the diotic vs. room-matched IC conditions (section Binaural Parameters and Level Mismatch, Figure 7) indicated a high correlation across subjects in both conditions, i.e., individuals exhibiting a high mismatch in the diotic condition most often also show a high mismatch in the room-matched IC condition. This provides further evidence that the individually reported mismatch is an individual treat, where the exact distribution of the internal spatial impression as controlled by the IC only exerts a small influence. Other factors (e.g., the individual’s ability to utilize binaural cues for better speech recognition under spatial talker-interferer conditions, cf. section Binaural Parameters and Level Mismatch) do not appear to have a stronger loading on the individually reported mismatch, thus making a prediction of this individual treat difficult. In other words, matching the IC during headphone presentation consistently reduces the size of the mismatch, while the general size of the mismatch is individual and determined by other factors that we could not identify in the present study in spite of an extensive auditory characterization of the subjects. Hypothesis H3 (individual markers of binaural hearing influences mismatch) thus has to be rejected.

To test hypothesis H2b, i.e., the influence of apparent source width on the mismatch, the relation between apparent source width and IC was analyzed in section Apparent Source Width and the Level Mismatch (Figure 8) for the non-anechoic rooms. With the broadband stimulus, the IC of the headphone presentation hardly affects the mismatch, but very clearly the average apparent source width rating. With the narrowband stimuli, the average apparent source width judgments are very consistent with the mismatch results (diotic vs. room-matched IC) – a “smaller” apparent source width as compared to the loudspeaker was associated with an increase of the mismatch by 3–5 dB (cf. Figures 5, 8). As for the mismatch, virtually no difference between average source width ratings was seen between the room-matched and uncorrelated conditions. On the individual level, no significant correlation between rated apparent source width and the loudness mismatch were observed. This can probably be attributed to the large variance in the apparent source width data, which may be caused by the rather hard task of comparing the perceived source width of a loudspeaker presentation occupying a certain part of auditory space around the loudspeaker with a headphone presentation that is most probably perceived as distributed somewhere in and around the head. The subjects may have had different internal interpretations of the apparent source width that could lead to much different results in the present experiment, e.g., the estimated absolute size of the source or its angular extent around the head. Also, the rather short stimuli of 1 s may have increased the difficulty of getting a feeling for the spatial characteristics of the different presentation modes.

Altogether, the present data support the hypotheses H2b that the mismatch can be reduced by adapting the interaural coherence during headphone to that with loudspeaker presentation, which also led to similar apparent source width judgments with loudspeaker and headphone presentation. This holds especially for narrowband stimuli in non-anechoic rooms, where diotic headphone presentation elicited a significant mismatch in most cases. With broadband stimuli, appropriate but weaker trends were also visible. Our data generally show that the mismatch is smaller with broadband stimuli, as is the influence of binaural parameters in headphone reproduction on the mismatch in general. We interpret the results as strong indicators of an influence of spatial perception on the mismatch. It cannot be finally concluded from the present data that a difference in spatial perception such as apparent source width, is the cause for a mismatch. However, in previous studies more spatially accurate headphone reproduction methods could avoid mismatch completely (Völk and Fastl, 2011; Brinkmann et al., 2017). While the apparent source width (cf. Rudmose, 1982) is one perceptual attribute of a plausible spatial perception, the present results show that eliciting the same apparent source width in headphone and loudspeaker presentation does not completely avoid the occurrence of a mismatch, especially when considering that the perception of source widths may differ fundamentally between loudspeaker and (unexternalized) headphone presentation. Similarly, the difference in spatial perception is even more different with monaural headphone presentation – which probably explains the difference to the mismatch seen with diotic headphone presentation that exceeded the common size of binaural loudness summation. In addition to the apparent source width, further perceptual attributes like the perceived externalization and distance, location, visual, and other multi-modal cues probably have to be adjusted correctly such that the mismatch disappears in a direct comparison, if the spatial perception is the dominant cause. Future studies should therefore examine the influence of more perceived spatial parameters on the loudness mismatch between headphone and loudspeaker presentation.

### Implications for Headphone Studies and Hearing Aid Fitting

The results presented in this study indicate that

1. A substantial level difference at equal loudness up to 15 dB exists for monaural presentation at ear-level vs. loudspeaker presentation to both ears in basically all conditions.
2. The interaural coherence in binaural ear-level presentation (and corresponding apparent source width) has a moderate influence of up to 5 dB on the mismatch in non-anechoic rooms. This effect vanishes in anechoic environments.
3. Small acoustic and/or visual changes in an anechoic reference environment (OL anechoic vs. AC anechoic) exert a moderate effect up to 5 dB on the recorded loudness mismatch, whereas not such a large effect of the respective reference room employed is observed across listening rooms with some reverberation (OL earpiecelab vs. AC teakitchen).

These findings – even though not completely explainable by the yet limited amount of parameter variations performed in this study – have already notable consequences whenever an implication for experiments in the free field has to be drawn from a condition with ear-level hearing devices or vice versa.

For hearing aid fitting, for example, diagnostic and prescriptive measurements (including loudness judgments) are most often performed independently for both ears using headphones, whereas the verification of the fit is performed for loudspeaker-like sources listened binaurally. Hence, the expected value of the loudness difference for monaural vs. binaural presentation and the frequency dependence of the mismatch across different IC conditions might provide a level correction value for the prescriptive “first fit” settings of the hearing device. However, the large variability in the mismatch across normal-hearing subjects and across the two anechoic rooms in this study would lead to the recommendation to be careful about using anechoic rooms for hearing aid fitting. Moreover, extensive fine-tuning should be performed with the hearing-impaired user of the hearing device, who might even show a much higher variability in binaural loudness summation especially for broadband sounds (Oetting et al., 2016).

For headphone studies, virtual acoustic reality is often aimed for by presenting sound signals *via* headphones that should reflect as closely as possible the individual’s perception (including loudness perception) in the free field. In applications of augmented reality, sounds from the free field and from ear devices are combined in order to enhance the free-field sound with added virtual sound. It is obvious that loudness perception from those two parts shall be matched. In order to minimize any loudness mismatch, narrowband stimuli should be used with the appropriate interaural coherence and special care has to be administered if non-anechoic conditions are employed. The present results further imply that in general a correct spatial perception of virtual sound sources is required to establish the same loudness at equal level.

## Conclusion

- The loudness comparisons in headphones and loudspeaker presentation in various environments employed here were combined with individual recordings of the HRTF and HpTF. This allowed for a careful and individual post-hoc quantification of the level mismatch at the eardrum across conditions that exhibit the same loudness.
- A substantial mismatch exists with a high variability across conditions and subjects which is strongly influenced by the presentation mode (monaural vs. binaural headphone presentation with a varying interaural coherence) and by the room acoustic conditions for the loudspeaker presentation. Remarkably, even differences between the anechoic rooms across sites using the same set of subjects were detected that may be due to small, but yet not explainable differences in room acoustics or non-acoustic factors. Such differences across sites did not occur for the tested non-anechoic rooms. Hence, the non-conclusive findings from the literature appear to be related to the experienced disparity between headphone and loudspeaker presentation, where even small differences in (anechoic) room acoustics significantly change the perception and response behavior of the subjects.
- The difference between monaural and binaural presentation during headphone comparisons yields an effect of 10 dB that goes beyond usual values for binaural loudness summation, while another difference of up to 5 dB occurs between diotic, dichotic, and room-matched interaural coherence during headphone presentation. A room-matched interaural coherence reduces the mismatch with respect to diotic presentation in non-anechoic rooms, but does not completely eliminate it.
- Individual factors like loudness summation appear to be only loosely connected to the observed mismatch, i.e., no direct prediction of the mismatch is possible from individual binaural loudness summation.
- Apparent source width coincides well with the differences in IC across diotic, room-matched, and dichotic conditions that do, however, not predict the loudness mismatch in a satisfactory way for broadband stimuli. Hence, other possible perceptual factors like, e.g., perceived distance, size, location; visual, and other multi-modal cues should be considered in future studies.
- Further experiments will have to gain a more detailed understanding by avoiding some of the shortcomings of the current study, i.e., individual binaural synthesis to produce the correct spatial image already during headphone presentation, and a better control of non-acoustic factors like visual cues provided during the experimental conditions.

## Data Availability Statement

The datasets presented in this study can be found in online repositories. The names of the repository/repositories and accession number(s) can be found at: https://doi.org/10.5281/zenodo.4153118 (HRTF and HpTF data) and https://doi.org/10.5281/zenodo.4153154 (equal-loudness levels at eardrum, further psychoacoustical results).

## Ethics Statement

The studies involving human participants were reviewed and approved by Ethik-Kommission an der Medizinischen Fakultät der RWTH Aachen, EK 351/17. The patients/participants provided their written informed consent to participate in this study.

## Author Contributions

FD, MK, JL-B, MV, and BK designed the general experiment. FD implemented the psychoacoustic experiments and the HRTF/HpTF measurements in Oldenburg. MK implemented the HRTF/HpTF measurements in Aachen. FD and JL-B conducted the measurements. FD and MK evaluated the data and prepared the figures. All authors contributed to the article and approved the submitted version.

### Conflict of Interest

The authors declare that the research was conducted in the absence of any commercial or financial relationships that could be construed as a potential conflict of interest.

## Footnotes

^1^Kohnen, M., Denk, F., Llorca-Bofí, J., Kollmeier, B., and Vorländer, M. “Cross-site investigation on head-related and headphone transfer function measurements: Implications on loudness balancing,” to be submitted to Acta Acustica.

^2^Additional corrections in AC_teakitchen: 250 Hz: +0.8 dB; 1 kHz: +2.5 dB; 4 kHz: +1.5 dB.

^3^Note that this analysis grouped the cross-site subjects that participated in all rooms with the other subjects participating only at both rooms of one site. Although data independence between factors is not strictly given, we expect no effects on the statistical outcomes. This is supported by the observation that equivalent outcomes were obtained by running a repeated-measures ANOVA on the results with cross-site subjects only.
